# Prescription Writing Trends of Antihistamines at the University Health Centre

**DOI:** 10.4103/0250-474X.56037

**Published:** 2009

**Authors:** Anil Kumar, Beenta

**Affiliations:** University Institute of Pharmaceutical Science, Panjab University, Chandigarh-160 014, India

**Keywords:** Antihistamines, cetirizine, chlorpheniramine maleate, diphenhydramine HCL, prescription monitoring, promethazine

## Abstract

The aim of the present study was to establish antihistamines drug prescribing pattern in order to improve the rational prescribing of antihistamines by physicians at Panjab University Health Centre. The study was performed in between the months of November 2005 to April 2006. Five hundred out patients were monitored and data was collected on WHO-based prescription-auditing performa. Demographic analysis of this prospective study revealed that out of the 500 patients, 293 (58.6 %) were male and 207 (41.4 %) were female and maximum patients were in the age group of 21-40 (34.8 %). Chlorpheniramine maleate (235 prescriptions) was the highest prescribed among antihistamine prescriptions (36.89 %) followed by diphenhydramine hydrochloride (186 prescriptions, 29.19%), cetirizine (175 prescriptions, 27.47 %) and promethazine (41 prescriptions, 6.4%). In comparison to generic drugs (169 prescriptions, 26.54%), branded were more prescribed at PUHC. Majority of antihistamines were in form of tablets (414 prescriptions, 64.99%) followed by liquid formulations (195 prescriptions, 30.61%) and injections (28 prescriptions, 4.40%). The average cost of different antihistamine drugs prescribed was as follows: diphenhydramine hydrochloride Rs. 34.74 followed by promethzine Rs. 22.46, chlorpheniramine maleate Rs. 15.30, and cetirizine Rs. 13.50. Average numbers of drugs prescribed per prescription were 1.27. The average consulting and dispensing time was 4.82 and 3.56 min, respectively. Out of the 500 university patients, 258 (51.6%) had the knowledge regarding the medication prescribed and 242 (48.4%) were unaware of the medication prescribed.

Antihistamines are used widely to treat the symptoms of various allergic reactions like allergic rhinitis and utricaria and hay fever etc. They are used for the prevention of motion sickness and morning sickness as well. Their efficacy, tolerance and safety in humans have been widely established[1] and hence they make up one of the largest groups of pharmaceutical agents used worldwide. Besides, their different therapeutic uses, the first generation antihistamines are relatively more sedative and block autonomic receptors. Second generation, comparatively less sedating due to their less distribution into the central nervous system. Evidence showed significant quantitative differences in the prescribing patterns of antihistamines between different countries[2]. Present investigation is a continuation of our ongoing work; where antihistamines were among major prescribed category of drugs. So, with this background the present study was designed to provide insights on the prescribing trends for antihistamines in order to improve the rational and cost effective use of antihistamine drugs and comment upon on any lacunae in their prescribing at the Panjab University Health Centre (PUHC).

The PUHC also known as Bhai Ghanayia Ji Health Centre, located centrally in the Panjab University campus, Sector 14, Chandigarh, India. It serves the health care needs of students and employees of Panjab University (teachers, nonteaching members) and their dependents. The drugs are dispensed through hospital pharmacy by pharmacists. The hospital procures drugs on annual basis. Drug purchase committee approves the drug list. There are 11 physicians, 6 pharmacists, 6 nurses and 3 clerks and one chief pharmacist who control and maintain the inventory stock of drugs at PUHC. The patients visiting PUHC have their own individual health cards and those who do not have one, use prescription slips (mainly for University students and hostellers having identity card). WHO based prescription-auditing proforma[3] was used to fill the demographic-related (name, age, sex and disease diagnose), drug-related (name of drugs, category, individual/combination, generic/branded, medicine dispensed or not and cost of medicine) and patient-related information (dispensing time, consultation time, pharmacist instruction and patient awareness on medication).

A pilot study was conducted at PUHC by previous consent of Chief Medical Officer to collect information from patients though a chance random method. A total of 500 patients were monitored during OPD timing 9.00 am to 12.30 pm (morning session) and 5.00 pm to 6.00 pm (evening session) from November 2005 to April 2006. Only antihistamine prescriptions were included in the study. Incomplete prescriptions or non-respondent patients were excluded from the study.

Study revealed that name (500, 100%) and age (394, 78.8%) and sex (500, 100%) were reported in majority of the prescriptions. Most of the patients were in the age group of 21-40 (174, 34.8%) fig. 1. Demographic data also indicated that out of the 500 patients, (293, 58.6%) were male and (207, 41.4%) were female. Provisional disease diagnose was reported in majority of the prescriptions (415, 65.14%).

**Fig. 1:**
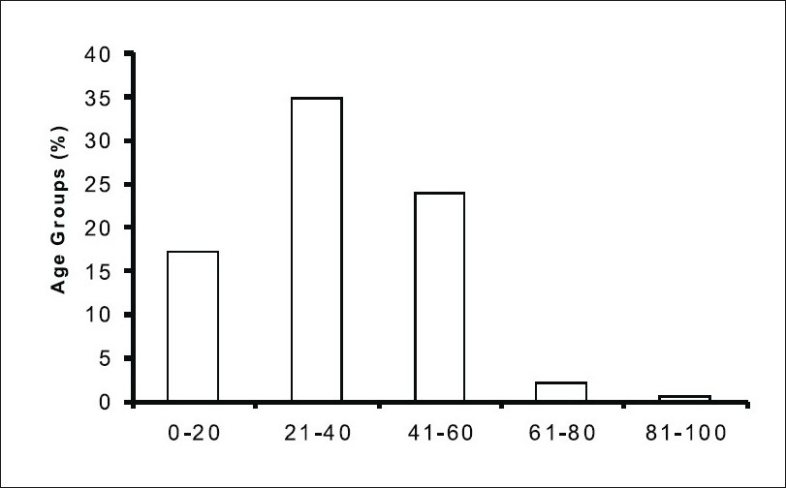
Antihistamines prescribed to different age group at PUHC. The figure shows the plot between percentages of antihistamines prescribed to different age group of the patients at PUHC.

In the overall 500 prescriptions, different antihistamines were prescribed (637 times) and among these, chlorpheniramine maleate (235, 36.89%) was highly prescribed followed by diphenhydramine HCl (186, 29.19%), cetirizine (175, 27.47%) and promethazine (41, 6.4%). These antihistamines were prescribed for simple cough/cold, fever, throat infections, pain, tonsillitis, pharyngitis, respiratory infection, allergic, and skin infection (Table 1). Out of 637 antihistamines, 26.54% and 73.46% were prescribed as generic and branded, respectively. Tablet dosage form 414 (64.99%) was more prescribed followed by liquids 195 (30.61%) and injections 28 (4.40%) at PUHC. The average number of antihistamine drug prescribed per prescription was 1.27. Majority of the drugs were dispensed from PUHC dispensing counter 510 (80.06%). The average costs of different antihistamines prescribed were diphenhydramine hydrochloride Rs. 34.74 followed by promethzine Rs. 22.46, chlorpheniramine maleate Rs. 15.30 and cetirizine Rs. 13.50 and (Table 2).

**TABLE 1:** AVERAGE COST OF ANTIHISTAMINICS AS PER MIMS

Antihistaminic Prescribed	Brand name	Average cost (In rupees)*
Diphenhydramine HCL	Lupihist syrup®, Benadryl syrup®	34.74
Promethazine	Phenargan syrup®	22.46
Chlorpheniramine maleate	Medler, Chericof syrup®, Cosome syrup®, Phensedyl syrup, Cheston syrup®, Ambrol plus syrup®, Cozyplus syrup®	15.30
Cetrizine	Cetirizine, Viscodyne syrup®	13.50

* MIMS Vol. 28 (12), December, 2008

**TABLE 2:** AVERAGE COST OF THE ANTIHISTAMNICS IN DIFFERENT PROBLEMS

Diseases	No of Prescriptions N (%)	Average Cost (in Rupees)*
Cough/Cold	175 (35)	26.05
Fever/infection	146 (29.2)	25.40
Pain	52 (10.4)	23.95
RTI	37 (7.4)	23.74
Pharyngitis	25 (5.0)	18.31
Tonsillitis	30 (6.0)	14.62
Allergy/Skin infection	20 (4.0)	14.35
Others	15 (3.0)	15.66

* MIMS Vol. 28 (12), December, 2008

The average consulting time was 4.82 min and the average dispensing time was 3.56 min. The patients were given only oral instructions regarding their dosing schedule. No written instructions were given to patients. This is one of the lacunae in the existing dispensing practice of the pharmacist. Out of the 500 patients, 258 (51.6%) had the knowledge regarding the medication prescribed and 242 (48.4%) were unaware of the medication prescribed.

The prescription-based survey is considered to be one of the most effective methods to assess and evaluate the prescribing attitude of physician and dispensing practice of pharmacists[4,5]. The demographic parameters such as age, sex are important in deciding dose, duration and dosage forms particularly in the case of pediatric population[7]. In the present study, name and sex of the patients were mentioned in all patient health record and age was reported only on 78.8% (394) of cases. Without proper age and appropriate sex, it is difficult to judge rationality of the prescription. Provisional disease diagnose was reported in majority of the prescriptions. Different antihistamines (mild or moderate sedative and or non sedative) were prescribed at PUHC. These antihistamines were used in combinations such as chlorpheniramine maleate with diphenhydramine hydrochloride, chlorpheniramine maleate with cetirizine or diphenhydramine hydrochloride with cetirizine or chlorpheniramine maleate with promethazine, although the second generation antihistamines (cetrizine) were less prescribed in PUHC. Tablet dosage form was more prescribed that could be due to adult patient population. The average number of drugs per prescription was found to be 1.28. This is a trend towards rational prescribing as it is preferable to keep average the number of drugs per prescription as low as possible to avoid drug interactions[8,9].

The average cost of antihistamines prescribed was found to be greater for diphenhydramine hydrochloride (Rs. 34.74), followed by promethzine Rs. 22.46, chlorpheniramine maleate Rs. 15.30, cetirizine Rs.13.50 (Table 2). Moreover, the cost of antihistaminic for cough and cold was Rs. 26.05 followed by fever Rs. 25.40, pain Rs. 23.95, respiratory tract infection Rs. 23.74, pharyngitis Rs. 18.31, tonsillitis Rs. 14.62 and allergy and skin infection Rs.14.35 (Table 1). However, costs of the prescriptions for these problems may be further reduced by formulating treatment guidelines for PUHC. Present study highlights that proper guidelines for antihistamines prescribing are required to rationalize the cost effective treatment by antihistamines.

Patient care indicator suggests that physician give sufficient time to patients in diagnosis at UHC in spite of heavy rush at morning and evening OPD. Similarly, pharmacists at dispensing counter dispensed the drugs with oral instructions only and there were no written instructions provided to the patients. So, lacunae in the existing practice can be improved by providing written instructions to patients. Improvement in the existing practice would definitely improve patient compliance for effective drug use[10]. Besides, upon patient interview, majority of the university patients or their dependent were aware of the type of medications prescribed.

Based on the above pilot study, an interventional study has been planned in order to improve prescribing patterns of antihistamines at PUHC. Interventional study would be helpful in improving safety, pharmacoeconomics of antihistamine prescribing at PUHC.
